# A Potentially Misleading Name: *Staphylococcus argenteus* in an Urosepsis: A Case Report

**DOI:** 10.1155/crdi/2880273

**Published:** 2025-11-07

**Authors:** E. Maillart, N. Yin, M. Dumitru, Y. Hasnaoui, M. T. Talpos, B. Mahadeb, P. Clevenbergh

**Affiliations:** ^1^Infectious Diseases Clinic, Brugmann University Hospital, Free University Brussels, Brussels, Belgium; ^2^University Laboratories Brussels (LHUB), Free University Brussels, Brussels, Belgium; ^3^Internal Medicine Department, Brugmann University Hospital, Free University Brussels, Brussels, Belgium; ^4^Intensive Care Unit, Brugmann University Hospital, Free University Brussels, Brussels, Belgium; ^5^Brugmann Laboratory, Brugmann University Hospital, Free University Brussels, Brussels, Belgium

## Abstract

*Staphylococcus argenteus*, a recently recognized species within the *Staphylococcus aureus complex*, shares numerous virulence traits with *S. aureus*. While *S. argenteus* typically lacks pigmentation and exhibits greater antibiotic susceptibility compared to *S. aureus*, it can cause severe infections, including bacteremia. Clinicians can be misled by its name and may not give it the necessary attention. It may also be misidentified as a *S. aureus* by the microbiology lab. We present the case of a 56-year-old man with a complex medical history who developed a polymicrobial urosepsis involving *S. argenteus*. It was initially misinterpreted as a contaminant in urinary samples by an external laboratory. The patient subsequently developed septic shock and was admitted to intensive care unit. The blood cultures confirmed *S. argenteus* associated with *E. coli* and *K. pneumoniae* bacteremia. He was successfully treated with high-dose intravenous floxacillin and oral ciprofloxacin. Whole-genome sequencing confirmed the isolate as *S. argenteus* (ST2250). This case underscores the diagnostic and clinical challenges posed by *S. argenteus*, particularly in regions where its prevalence is low. *S. argenteus* harbors the majority of *S. aureus* virulence genes and causes a comparable spectrum of disease. Epidemiological data indicate regional differences in its prevalence and clinical impact. Some studies report lower virulence while others suggest worse outcomes. The case illustrates the importance of accurate identification using techniques like MALDI-TOF MS and whole-genome sequencing. Clinicians and microbiologists should remain vigilant and consider *S. argenteus* as a pathogen, warranting appropriate antimicrobial therapy and clinical attention. Differentiating it from *S. aureus* might be relevant for guiding therapy, surveillance, and infection control, particularly given emerging reports of methicillin-resistant *S. argenteus* strains.

## 1. Introduction

The genus *Staphylococcus* (comprising 71 currently recognized species) consists of facultatively aerobic, Gram-positive cocci that typically appear in clusters under microscopic examination. The strains are categorized into two main groups: coagulase-positive and coagulase-negative staphylococci. The ability to clot animal plasma is closely linked to their pathogenic potential. Based on whole-genome sequencing (WGS), the 13 coagulase-positive staphylococcal species can be divided into three clades, among which the *S. aureus* complex (SAC) is the best-known human pathogen. The SAC itself includes *S. aureus stricto sensu*, *S. argenteus*, *S. schweitzeri*, *S. roterodami*, and *S. singaporensis*, which are phylogenetically distinct species [1, 2]. Among these, *S. schweitzeri* does not appear to be pathogenic to humans but has the potential to become a zoonotic agent [3]. It has already been identified in nonhuman primates closely related to humans [4]. *S. argenteus*, which is both coagulase- and catalase-positive, lacks pigmentation when cultured. However, it is frequently misidentified as *S. aureus*, from which it was differentiated in 2015 [5]. There is ongoing debate regarding the clinical importance of distinguishing *S. argenteus* from *S. aureus*. Evidence suggests that *S. argenteus* strains possess fewer virulence factors, such as PVL toxin, and show lower levels of antimicrobial resistance compared with *S. aureus stricto sensu*. The severity of infections caused by *S. argenteus* may also be lower [6]. Whether the clinical presentations of diseases caused by both species fully overlap remains to be determined. Nonetheless, it is important for clinicians to recognize that *S. argenteus* should not be dismissed as a nonpathogenic staphylococcus [7]. We report the case of a patient with *S. argenteus* bacteremia associated with a long-term indwelling urinary catheter.

## 2. Case Report

**Figure 1:**
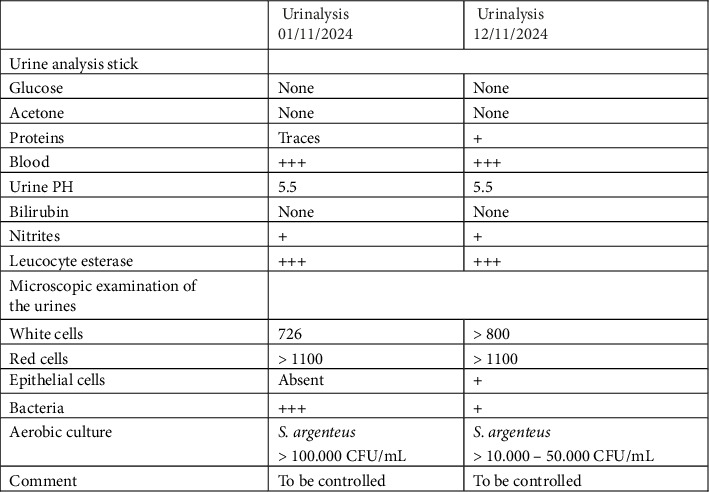
Urinalysis showing *S. argenteus* disregarded as a contaminant. S: susceptible; I: susceptible to increased dosage; R: resistant.

**Figure 2:**
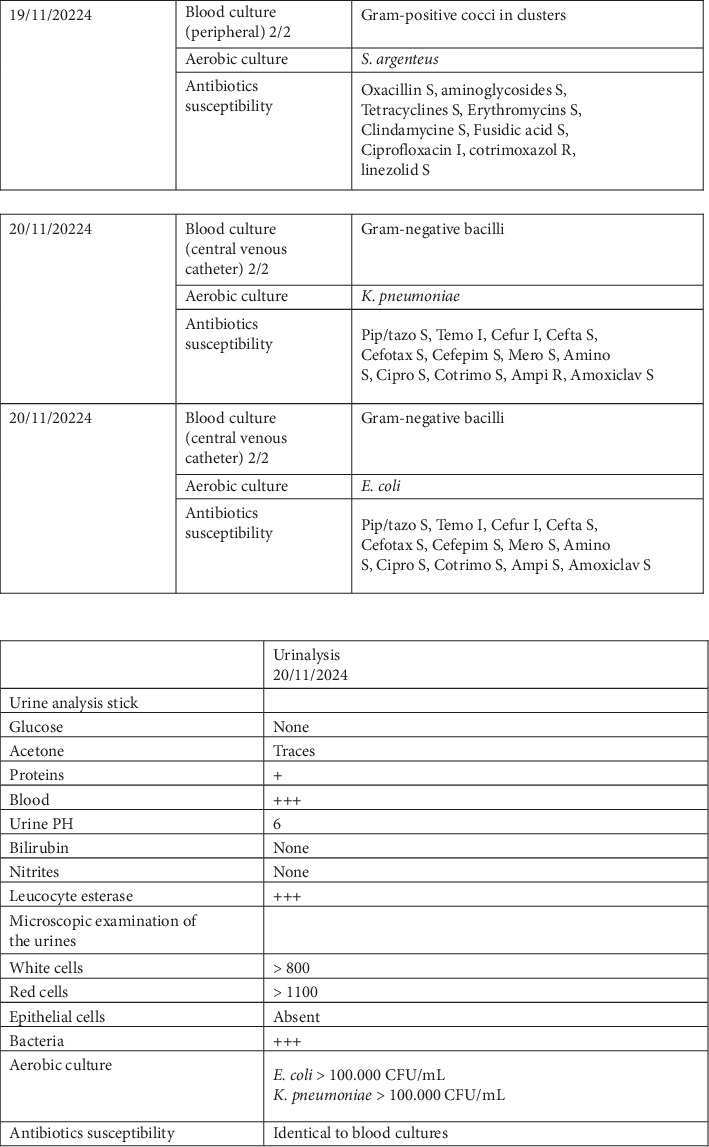
Blood cultures growing *S. argenteus* and another set of blood culture growing *E. coli* and *K. pneumoniae*. Urinalysis growing the same germs. S: susceptible; I: susceptible to increased dosage; R: resistant.

This 56-year-old man was admitted in October 2024 with severe acute hemolysis and dehydration. His medical history included Niemann–Pick type C disease, chronic renal failure, von Willebrand disease, Type 2 diabetes mellitus, hypertension, former cocaine abuse, and severe depression. His chronic medications included antihypertensives, antidiabetic agents, and miglustat for his lysosomal storage disorder. The patient was admitted to the ICU on 25/10/2024 for the management of thrombocytopenia and metabolic disturbances. A bladder catheter was inserted the same day (25/10/2024). On 6/11/2024, the patient accidently removed the urinary catheter, resulting in macroscopic hematuria. A triple-lumen catheter was subsequently inserted to allow continuous bladder irrigation. On 01/11/2024, a urinalysis performed by an external laboratory revealed 726 WBC/mL, > 1000 RBC/mL, and 100,000 CFU/mL of *S. argenteus*. The isolate was dismissed as a contaminant, and no antimicrobial susceptibility testing was performed, with the laboratory recommending a repeat urinalysis. On 12/11/2024, a repeat urinalysis again isolated *S. argenteus*, accompanied by the same laboratory comment (Figure 1). On 20/11/2024, the patient was readmitted to the ICU with septic shock, presumed to be of urinary tract origin, and was empirically treated with piperacillin–tazobactam plus a single dose of amikacin. At LHUB-ULB, laboratory investigations revealed WBC 6770/μL, neutrophils 98%, CRP 166 mg/dL, PCT >  99.99 μg/L, lactate 6.5 mmol/L, eGFR 19 mL/min, ALP 424 U/L (< 129), γGT 317 U/L (< 71), total bilirubin 5.6 mg/dL (< 1.2), and glucose 202 mg/dL. A blood culture collected on 19/11/2024 grew *S. argenteus*, previously detected in the urine (Figure 2). Antimicrobial susceptibility testing (AST) was performed using the VITEK 2 AST-P652 system (BioMérieux, Marcy l'Etoile, France) and interpreted according to EUCAST version 13.0. *S. argenteus* was susceptible to all antistaphylococcal agents tested, except cotrimoxazole. A second set of blood cultures (20/11/2024) grew *Escherichia coli* (wild type) and *Klebsiella pneumoniae* (wild type), which were also detected in urine samples (Figure 2). On 24/11/2024, antibiotic therapy was switched to amoxicillin–clavulanate, to which *E. coli* and *K. pneumoniae* were susceptible. Subsequently, the infectious diseases specialist modified treatment to high-dose intravenous flucloxacillin for *S. argenteus* and oral ciprofloxacin for *E. coli* and *K. pneumoniae*, administered for 14 days. Follow-up blood cultures collected on 26/11/2024 remained sterile. Identification of the three bloodstream isolates was confirmed by MALDI-TOF mass spectrometry (Sirius, MBT IVD reference library version 2023, Bruker Daltonics, Bremen, Germany). The *S. argenteus* strain underwent WGS. DNA extraction was performed using the EZ1 & 2 Virus Mini Kit v2.0 (Qiagen, Hilden, Germany) and the EZ2 Connect MDx system (Qiagen). Genomic DNA was enzymatically fragmented and prepared into an Illumina-compatible library using Revelo DNA-Seq for MagicPrep NGS (Tecan, Männedorf, Switzerland). Sequencing was carried out on an Illumina MiniSeq platform (San Diego, USA) using the MiniSeq Mid Output Kit (300 cycles) in 2 × 150 bp paired-end mode. De novo genome assembly was performed using the Velvet algorithm within Ridom SeqSphere+ version 10.0.5 (Ridom GmbH, Münster, Germany). Species confirmation was obtained using NCBI RefSeq Masher [8]. The top three matches corresponded to *S. argenteus* with matching scores between 299 and 388/400, distance < 0.01, and *p* value = 0, whereas the next 17 matches were *S. aureus* with matching ≤ 65/400 and distance > 0.07. Multilocus sequence typing (MLST) using the *S. aureus* scheme identified sequence type ST2250, confirming the isolate as *S. argenteus*. The assembled genome was deposited in the National Center for Biotechnology Information (NCBI) under the BioSample accession number SAMN47436392. One month later, the patient's neutrophil count, inflammatory markers, renal function, and liver enzyme levels had normalized. Follow-up urinalysis, performed at the external laboratory on 29/11/2024, showed sterile results.

## 3. Discussion

Infections caused by *S. argenteus* have been predominantly reported in Asia, South America, and Australia, while cases from Western Europe remain rare. In a German study, *S. argenteus* was found to be 100 times less frequent than *S. aureus* [7]. In Belgium, it represents approximately 1 in 500 isolates [9]. However, in certain regions, *S. argenteus* is significantly more prevalent. In a Thai study, nearly 20% of infections attributed to the SAC were in fact caused by *S. argenteus* [6]. In the same German study, patients infected with *S. argenteus* were less likely to receive initial flucloxacillin therapy than those infected with *S. aureus*, possibly because clinicians were unaware of the pathogenic similarities between the two species. The staphyloxanthin operon, a key virulence factor in *S. aureus* [10], is absent in *S. argenteus*, explaining its whitish, nonpigmented appearance on culture media. However, most of the core virulence genes identified in *S. aureus* are also present in *S. argenteus* [11, 12]. Indeed, over 75% of the virulence-associated genes found in *S. aureus* have been recovered in *S. argenteus* [13]. The ESCMID Study Group on Staphylococci and Staphylococcal Diseases has recommended including the phrase “member of the SAC” when reporting *S. argenteus*, or even not distinguishing it in routine diagnostic results [14]. The clinical spectrum of diseases caused by the two species appears largely similar, as illustrated in the German case series, which included infections associated with foreign bodies. *S. argenteus* can cause bloodstream infections, skin and soft tissue infections, bone and joint infections, endocarditis, and food poisoning. However, studies comparing its pathogenicity with *S. aureus* have yielded conflicting results [6, 15]. In a Taiwanese study, patients with *S. argenteus* bacteremia had a 1.8-fold higher risk of death compared with those infected by *S. aureus*. *S. argenteus* infections were more often polymicrobial and healthcare-associated and were linked to thrombocytopenia, lower respiratory tract infections, and short-term mortality [15]. Conversely, in a Thai study, *S. argenteus*-infected patients were less likely to develop respiratory failure and were equally likely to experience septic shock or 28-day mortality compared with *S. aureus*-infected patients. *S. argenteus* was also more susceptible to antibiotics and carried fewer toxin genes (pvl and seb) than *S. aureus* [6]. However, both studies included relatively small sample sizes, limiting the ability to fully distinguish between the two species. In a Hong Kong case series, 11 *S. argenteus* strains (4% of SAC bacteremia cases) were methicillin-sensitive and generally susceptible to standard antistaphylococcal antibiotics. No Panton-Valentine leukocidin (PVL) or toxic shock syndrome toxin-1 (TSST-1) was detected, though multiple SE genes—associated with food poisoning and toxic shock—were present. The authors concluded that, due to differences in antimicrobial susceptibility, resistance genes, and toxin expression, differentiating *S. argenteus* from *S. aureus* is clinically relevant. Methicillin-resistant (MR) *S. argenteus* strains, resistant to flucloxacillin, have been identified within collections of MR *S. aureus* (17 out of more than 4000 MRSA isolates) [16]. The authors of that study suggested that MR *S. argenteus* (MRSarg) should not be managed with the same infection control measures as MRSA, though no evidence currently supports such a distinction [16]. *S. argenteus* can be distinguished from *S. aureus* using MALDI-TOF MS [17, 18]. However, WGS provides superior accuracy and is considered the reference method [19]. In our case, the external microbiology laboratory initially dismissed *S. argenteus* isolates from urine samples, recommending repeat testing without performing antimicrobial susceptibility testing. This was unusual, as the laboratory typically provides susceptibility results for all uropathogens, regardless of whether they are deemed pathogenic or colonizing/asymptomatic. Subsequently, intensive care physicians adjusted antibiotic therapy to target Gram-negative organisms, overlooking specific treatment for *S. argenteus*. As reported in the literature, this oversight is more likely in regions such as Belgium, where *S. argenteus* remains rare. Our patient was initially treated with antibiotics that did not provide adequate coverage against *S. argenteus*. He also had a polymicrobial infection but ultimately recovered after receiving targeted antistaphylococcal therapy.

## 4. Conclusion

The *Staphylococcus aureus* complex comprises five recognized species, among which *S. aureus* and *S. argenteus* share comparable virulence profiles and overlapping clinical disease spectra. We describe a case of polymicrobial urosepsis involving *S. argenteus* in a patient with a chronic indwelling urinary catheter. The patient responded successfully to flucloxacillin therapy, although *S. argenteus* was initially dismissed as a nonpathogenic staphylococcal isolate. While it remains uncertain whether distinguishing *S. argenteus* from *S. aureus* has consistent clinical implications, clinicians should recognize *S. argenteus* as an emerging pathogen. Conflicting evidence exists regarding the relative severity of infections caused by *S. argenteus* compared with *S. aureus*. *S. argenteus* is generally susceptible to standard antistaphylococcal agents and harbors fewer toxin genes than *S. aureus*. Nevertheless, most virulence-associated genes are conserved across both species. Accordingly, *S. argenteus* should be managed with the same clinical diligence and therapeutic rigor as *S. aureus*. Microbiology laboratories are encouraged to include an interpretative comment when specifically identifying *S. argenteus*. MRSarg should be subject to the same infection control and decolonization measures as MRSA.
